# Security Cost Aware Data Communication in Low-Power IoT Sensors with Energy Harvesting

**DOI:** 10.3390/s18124400

**Published:** 2018-12-12

**Authors:** Xiaolin Fang, Ming Yang, Wenjia Wu

**Affiliations:** School of Computer Science and Engineering, Southeast University, Nanjing 211189, China; yangming2002@seu.edu.cn (M.Y.); wjwu@seu.edu.cn (W.W.)

**Keywords:** security cost, data transmission, energy harvesting, low-power

## Abstract

Security is a critical concern in low-power IoT (Internet of Things) wireless sensors because these resource constrained devices are easy to attack and meanwhile the energy constraint sensors will consume a lot of energy to run algorithms for security purposes. We study the energy efficiency data transmission problem in IoT sensors that use capacitors to harvest wireless energy while considering the energy cost for running security algorithms. Energy harvesting with capacitors has the characteristic that the energy harvesting rate varies over time, and it is getting slower and slower as the capacitor gets more and more wireless energy. This observation will result in a trade-off for data transmission in two ways: (1) dividing data into more number of packets, thus the sensors can receive wireless energy at a higher harvesting rate, but it will result in extra energy consumption; (2) dividing data into less numbers of packets—in this way, the sensor cannot utilize the high harvesting rate, but the extra energy cost is less. We studied two sets of this problem where the low-power sensors can harvest enough wireless energy or not, and give algorithms to transmit all the data or as much data as possible, respectively, while taking into account extra cost. The theoretical performance of the proposed algorithms is also analyzed. Both theoretical analysis and extensive simulations show that the proposed algorithms have good performance.

## 1. Introduction

Secure communication is a great challenge in IoT systems because the energy limited IoT sensors cannot provide computation intensive processing for high level security protection [1,2,3,4,5,6,7]. Even a lightweight computation process for a security function will consume a lot of energy in low-power sensors that have extremely limited battery energy. Thus, how to transmit data efficiently and in security should be carefully studied in such environment. Energy harvesting technology becomes an attractive approach to alleviate this problem because it can harvest environment energy and supply to the low-power sensors, and the sensors are expected to improve the security processing ability and live for a much longer time [8,9,10,11]. However, the harvested energy is still a rare resource considering that secure data transmission will consume the main part of the received energy.

**Figure 1:**
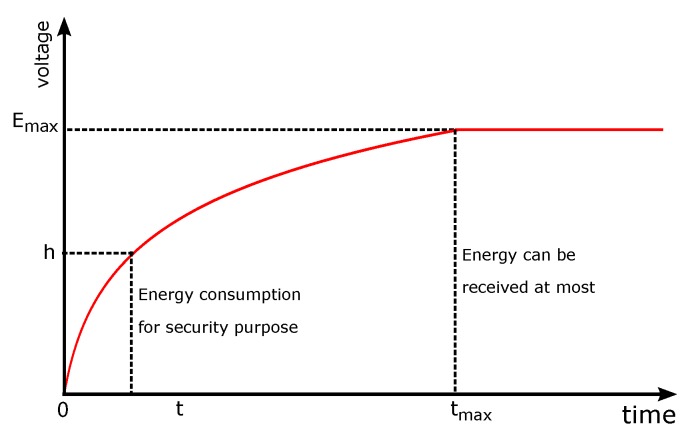
Energy harvesting process.

Recently, the technology of wireless power transfer has been used in the area of energy harvesting for IoT and cell networks [12,13,14,15,16,17]. In particular, the authors in [15,16] studied the problem of energy utility in energy harvesting devices and networks. They provided very good solutions that can derive performance within O(ϵ) of the optimal when the amount of batter that can be used is O(1/ϵ). In this paper, we study the problem of data transmission with a capacitor to supply wireless energy while taking into account extra energy consumption. Sensors that apply capacitors to receive wireless energy for data transmission receive a lot of researcher’s focus [18,19,20,21]. The capacitor can receive wireless energy from a wireless signal that is transmitted several meters away. Some researchers start to study the problems of data transmission in IoT devices that utilize capacitors to receive wireless energy [22,23,24]. How to improve the efficiency of the energy utilization is the main problem for sensors that receive wireless energy with capacitors. It has been found that the energy received by the capacitor increases slower and slower as that the sensor gets more and more wireless energy, that is, the energy harvesting rate becomes slower and slower over the energy receiving process [24,25,26,27,28]. Thus, the energy harvesting rate changes over time. When the capacitor is getting full, the energy receiving rate is becoming slower. As shown in Figure 1, the energy received over time is nonlinear and it is getting slower and slower. When energy received by the capacitor gets to $E_{max}$, then the capacitor almost cannot receive wireless energy any more.

With the purpose of achieving secure data transmission, the IoT sensors usually need to run encryption algorithms so that data cannot be stolen by a hostile attacker easily [29,30,31,32]. However, even a lightweight encryption algorithm will consume a lot of energy in low-power sensors with capacitors to receive wireless energy. Using Figure 1 as an example, let the energy that can be received by the sensor be $E_{max}$, and the extra energy cost for a packet be *h*; then, only the remaining energy $E_{max}-h$ can be used for data transmission. Thus, during the wireless energy harvesting time, the most efficient harvesting time [0,t] is used to receive the energy for running the lightweight encryption algorithm, while, in the remaining time, $\left[t,t_{max}\right]$ will receive the energy for data transmission. The energy harvesting rate is much lower in $\left[t,t_{max}\right]$ than that in [0,t] as shown in Figure 1; that is, it will take a much longer time to receive the same energy that can be received within [0,t] after time *t*. Based on this observation, given some amount of data, it is difficult to design a method to put the arrived data into some smaller packets and determine when to receive wireless energy and when to transmit data so as to improve the utilization of the harvested energy.

This paper will study the problem of how to improve the energy utilization considering the energy consumption that resulted from data transmission and security computation in IoT sensors that use capacitors to receive wireless energy. We try to give an optimization solution for data transmission so as to take full advantage of the nonlinear energy receiving characteristic. It assumes that IoT devices first need to harvest enough wireless energy and then they can start to run encryption algorithms and transmit data, and the sensor cannot receive wireless energy when it is transmitting packets. In some cases, the IoT sensors cannot always harvest wireless energy all the time because the wireless energy transmitter may be turned off. If there are some wireless energy receiving periods arriving at different times, how to transmit as much data as possible is a hard problem. The difficulty is that the energy receiving periods arrive over time, the energy harvesting rate changes over time, and different data blocks arrive over time; thus, the sensor cannot get all the information at the initial time. The following lists the contributions of this paper:

- We study the problem of energy utilization that resulted by data transmission in IoT devices that use capacitors to receive wireless energy. Different from existing studies, if the sensors use capacitors to receive wireless energy, the wireless energy receiving rate changes over time, and it gets slower and slower.
- If the IoT sensors do not have enough wireless energy receiving time, the minimum completion time problem, which transmits all the data, is studied. An online algorithm is provided and the competitive ratio is $\frac{n+3}{n+1}$.
- If the IoT sensors have enough wireless energy receiving time, the problem of transmitting the max amount of data is studied. Two cases for this situation where there is only one energy receiving period and there are multiple energy receiving periods are studied. Two competitive ratio online algorithms for both of these two cases are provided in this paper.

The rest of the paper is organized as follows: Section 2 gives the problem definition. Section 3 studies the problem of how to transmit all the data to minimize the total completion time. Section 4 studies the problem of how to transmit the data so as to maximize the amount of successfully transmitted data. Section 5 evaluates the performance of the provided algorithm in simulations. Section 6 concludes this paper.

## 2. Preliminary and Problem Statement

Our previous work [33] started earlier work to consider the difficulty of such problem. Because the energy receiving process with capacitor is nonlinear, it looks like a logarithmic function; we thus study the problem of data transmission considering that the wireless energy receiving rate changes over time. Let the energy receiving function be H(t); then, $H^{-1}\left(b\right)$ is the time that the sensor required to receive *b* units of energy. The function H(t) is nonlinear. We do not give the exact function because the algorithms we design later can solve the problem of whatever the energy function H(t) looks like as long as it is a convex function. That is, as long as the energy receiving rate is getting slower and slower, our designed algorithms can all work well. This paper assumed that the wireless energy receiving process is stable. This is reasonable because, in many applications, the energy transmitter and energy receiver do not need to move. A stable wireless energy receiving process can be realized in the situation that the distance between energy receiver and transmitter does not change.

Let the wireless energy receiving periods be represented as $E=\{E_{1},E_{2},\ldots ,E_{m}\}$, $E_{i}=\left[s_{i},e_{i}\right]$, where $s_{i}$ is the beginning time, and $e_{i}$ is the finishing time of the *i*th energy receiving period. If there are *n* blocks of data $D=\{D_{1},D_{2},\ldots ,D_{n}\}$ that should be transmitted, $D_{i}=\langle a_{i},b_{i}\rangle$, where $a_{i}$ is the time that the data block arrives, $b_{i}$ means that it will consume $b_{i}$ units of energy to transmit this block of data. If the IoT sensor can receive enough energy, it should find a method to transmit all the data so as to maximize the total completion time. Otherwise, if the IoT sensor cannot receive enough energy, it should find a solution to transmit the data so as to maximize the total amount of successful transmitted data. In this paper, we study the problem of transmitting as much data as possible, rather than the biggest number of data blocks. This is because, in many cases, we do not have to consider a block as a whole. We can divide the data block into many pieces, and each piece is useful—for example, if a sensor has a block of data that should be transmitted, and this data block consists of a sequence of temperature values with different times. Then, if there is no enough energy to transmit the entire data block, transmitting a sub-sequence of temperature values is still useful. It can still supply some information to the data center to make decisions. The length of the sub-sequence of temperature values can be regarded as the amount of data. $E_{1}$ to $E_{m}$ and $D_{1}$ to $D_{n}$ cannot be known in advance. Energy behavior prediction is a way to alleviate this problem. However, in this paper, we assume that the arriving information is random and irregular. This is reasonable because the future arriving data and energy receiving time are unpredictable in many applications. We use the denotes of $E_{i}$ and $D_{i}$ only for the definition of the problem. In practical situation, the information of $E_{i}$ and $D_{i}$ cannot be obtained initially. Therefore, we should design online algorithms. The online algorithms do not need to get the future arriving information (such as future arriving energy receiving periods, future arriving data and so on), and they can solve the problem based on the online arriving information and get an approximated solution. It is difficult to give such an optimal solution because the future arriving data information and energy receiving information are all unknown [34,35]. People usually use competitive ratios to evaluate how the designed online algorithms performs. It is a ratio that compares the result of the given online algorithm with an optimal result. The smaller the competitive ratio is, the better performance the online algorithm has.

**Figure 2:**
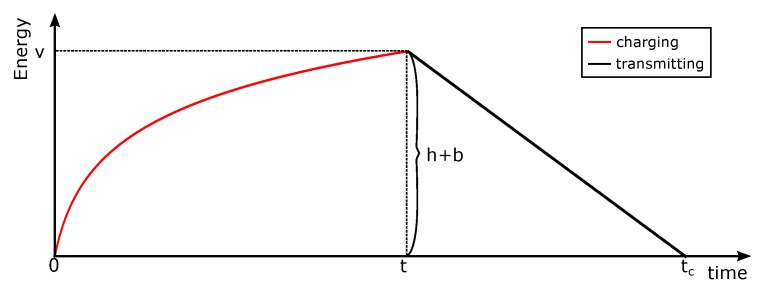
Energy receiving and data transmission process.

A packet usually consists of the packet header and the data needed to be transmitted. Let the size of the packet header be *h*, which means that it needs *h* units of energy to transmit the packet header. Let the data put in the packet be *b* and the transmission speed for a packet be *R*; then, it will take $\frac{h+b}{R}$ units of time to complete the transmission process. In this paper, the transmission cost is assumed to be a function of the data size and the packet header. It is assumed to be an average expected linear function. In a low collision environment, the function would be small. Furthermore, in a high collision environment, the transmission cost would be large. We only use this simple transmission cost for simplicity of performance analysis. If every exact retransmission is considered, the energy cost should be based on the practical situation; then, the performance bound of the online algorithm is very hard to analyze. It makes an assumption that the sensor will not receive wireless energy at the time it is transmitting packets. Let H(t) be the energy harvesting function; then, H(t)=h+b. An example is shown in Figure 2. In this example, it will take $\frac{h+b}{R}=t_{c}-t$ units of time to finish the transmission, and the completion time is represented as $t_{c}$ for this packet. In the example of Figure 2, the energy harvesting function is nonlinear, and the increasing rate of this function is becoming slower and slower. The energy consumption for data transmission is linear to the data size, that is, if the packet is larger, then it will consume more energy.

## 3. The Sensor Can Receive Enough Wireless Energy

In some situations, the sensor can receive enough wireless energy from an energy transmitter so that the sensor can transmit all the data blocks. This is the case where the energy transmitter and receiver are stable. Thus, let us consider the simple case where the energy receiving period is long enough, that is, $E_{1}=\left[0,+\infty \right]$. In this case, it needs to study how to transmit all the data as fast as possible, and we use the total completion time to evaluate the performance of the given algorithm. We first study the problem where there is only one huge data block, and then we continue to study the problem with multiple data blocks arriving over time. In the first sub-problem, an optimal solution can be found to transmit the huge data block in multiple smaller packets so that the total completion time is minimized. In the second sub-problem, we design an online algorithm to solve the problem, and prove that the total completion time is no $\frac{n+3}{n+1}$ times worse than any optimal solution.

### 3.1. Only One Huge Block of Data

Let us first consider the problem where there is only one huge block of data that should be transmitted. Assuming that the sensor has a huge block of data with size *B* needed to be transmitted, it should then determine how to send this block of data in one packet, or divide this block of data into multiple smaller packets and transmit the data multiple times. These two methods may result in different results. If the block of data is transmitted in one packet, then the sensor needs to transmit only one packet header. However, the wireless energy receiving rate is becoming slower and slower because of the characteristic of capacitors, the device will receive energy slower and slower; therefore, it will take much more time to harvest enough wireless energy to send this entire block of data. In another way, if the packet is divided into many smaller packets, the energy receiving rate is higher for smaller packets; however, it will introduce the cost of transmitting more packet headers, i.e., if the entire block of data is divided into *m* smaller packets, then the sensor needs to transmit *m* packet headers. Therefore, there is a trade-off between whether transmitting the block of data as a whole or dividing it into multiple segments and transmit it in many smaller packets.

**Figure 3:**
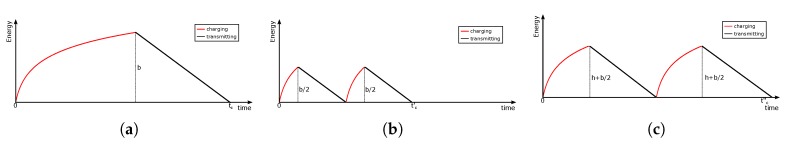
Find the minimum total transmission time. (**a**) transmit the data in one packet; (**b**) transmit the data in multiple smaller packets; (**c**) extra transmission cost of introducing the packet header.

Since the wireless energy receiving rate is getting slower and slower, an intuitive method is to fully utilize the received wireless energy as earlier as possible. An example is shown in Figure 3, where, if the sensor transmits a block of data in one packet (let the packet size be *b*) after the time it receives enough energy, then, as shown in Figure 3a, $t_{c}$ is the completion time for transmitting this block of data. However, if the sensor transmits this block of data in several smaller packets, then the $t_{c}^{\prime }$ is the completion time as illustrated in Figure 3b. It is obvious that the completion time $t_{c}^{\prime }<t_{c}$ in this example. The reason is that the energy receiving rate is getting slower and slower, and the capacitor can receive energy faster at the earlier time. Therefore, the sensor should use the received energy as soon as possible.

However, if a block of data is divided into many smaller packets, then it will introduce the cost of transmitting more packet headers. The packet header includes the IDs of the data transmitter and receiver and other information for the transmission protocol. Thus, transmitting the block of data in many packets may not be a good option. If this transmission cost is too heavy, it may increase the completion time for that block of data. In Figure 3c, two extra packet headers are transmitted and thus the completion time for such way $t_{c}{}^{\prime \prime }>t_{c}^{\prime }$. If the data is transmitted in even more packets, it will result in additional extra packet header cost, and thus a longer completion time.

If we want to minimize the completion time for this huge block of data with size *B*, as stated above, we should divide the data into a certain number of parts and transmit it in some number of packets but not too many packets so as to reduce the completion time. Then, we can use the following equation to represent this problem:

(1) $$\min\{m\cdot t+\frac{B+m\cdot h}{R}\},$$

subject to:

(2) H(t)=h+b,

(3) $$b=\frac{B}{m}.$$

In this problem, *B* is the amount of data that should be transmitted, *m* is the number of smaller data blocks that the data will be divided into. *b* is the data size in a packet. i.e., $b=\frac{B}{m}$, and *h* is the size of the packet header that should be transmitted. Assume that, if the sensor receives energy for a period of time *t*, then it will receive the energy to transmit a packet consisting of a segment of data with size *b* and a packet header with size *h*, that is, H(t)=h+b. Let *R* be the data transmission speed; then, total transmission time for the data is $\frac{B+m\cdot h}{R}$ and the total energy receiving time is m·t. Therefore, the completion time for this block of data with size *B* is $m\cdot t+\frac{B+m\cdot h}{R}$. The problem is to determine the period of time *t* that the sensor should take to receive wireless energy and then begin to transmit data. After *m* periods, the sensor will completely transmit the entire block of data with size *B*. The objective of this problem is to minimize the completion time for transmitting this entire block of data.

**Figure 4:**
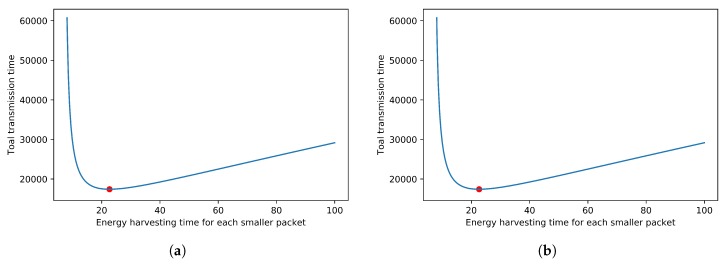
Find the minimum total transmission time. (**a**) header = 3. (**b**) header = 5.

If the sensor has enough energy receiving time, then the problem is how to minimize the total time for transmitting the entire data block with size *B*. We use an example in Figure 4 to show that there exists an optimal solution for this problem with only a huge data block and the dividing solution can be found through a binary search or Newton’s method. Figure 4 illustrates an example where the wireless energy receiving function is assumed to be $H\left(t\right)=\log_{2}\left(t+1\right)$, the data size is B=1000, and the transmission rate is set to R=1. The energy harvesting function is the energy that the capacitor will receive from the time the capacitor is initially empty. The data size is 1000, which is represented as 1000 units of energy for simplicity; in other words, it will take 1000 units of energy to transmit this block of data. In Figure 4, the *x*-axis is the time *t* that the sensor should take to receive energy and then transmit a packet immediately, and the *y*-axis is the total time it will take to complete the transmission for the whole block of data. From Figure 4a,b, it can be easily found that the minimum completion time is at the position of the red points. The length of the packet header for Figure 4a is 3, which means it will take three units of energy to transmit the packet header for each packet. The length of the packet header for Figure 4b is 5. For Figure 4a, the *x*-axis for the red point is about 23, and the *y*-axis is about 17,000, which means that if the sensor always takes about 23 units of time to receive wireless energy, then it uses all the received energy to transmit a segment of data in a packet, then it will take some periods to receive energy and transmit data. After some periods when the entire block of data with size B=1000 is completely transmitted, the completion time for transmitting this entire block of data is about 17,000 units of time. For Figure 4b, the size of the packet header is 5, the *x*-axis for the red point is about 90, and the *y*-axis is about 64,000. Similarly, it denotes that, if the sensor always takes about 90 units of time to receive energy, then uses all the received energy to transmit a segment of data in a packet, after some period, the completion time for transmitting this entire block of data with size 1000 is about 64,000 units of time. We let the energy receiving time (about 23) in Figure 4a and energy receiving time (about 90) in Figure 4b be the optimal energy receiving time $t^{*}$ that will be used in the later of this paper.

As shown in the example, it can be found that there is a point that can get the optimal result. Therefore, the problem can be solved by a binary search or Newton’s method. We can set a start point $t_{0}$ where $H(t_{0})>=h$, let $f\left(t\right)=m\cdot t+\frac{B+m\cdot h}{R}$, the Newton’s method can be solved using the following process until a sufficiently accurate value is obtained:

(4) $$t_{n+1}=t_{n}-\frac{f(t_{n})}{f^{\prime }\left(t_{n}\right)}.$$

**Definition** **1.** Let $b^{*}$ be the optimal amount of data that should be put in a packet, $t^{*}$ be the optimal energy receiving time the sensor should use to receive wireless energy and after that it starts to transmit a packet, and $E^{*}=h\left(t^{*}\right)=h+b^{*}$ be the optimal energy the sensor should receive and then begin to transmit a packet consisting of the packet header and the data payload.

### 3.2. Data Arrives at Different Times

Practically, data usually arrives over time. If a block of data comes, the sensor should determine sending this block of data at once or continuing to receive more energy so as to improve the energy utility. From Section 3.1, it can be found that there is an optimal time point that can minimize the completion time of data transmission when there is enough energy receiving time and the data arrives at time 0. However, because the data blocks arrive at different times, the information for the future coming data is unknown. Thus, it is difficult to determine when to receive energy and when to start to transmit data so as to minimize the total completion time for all the blocks of data.

We thus give an online algorithm that is provided in Algorithm 1 to solve the problem. The online algorithm always maintains a data list consisting of the data that has not been completely transmitted. Any arriving block of data is inserted into the list before those data blocks whose untransmitted data size is larger than the arriving data block’s size. The sensor always transmits the data block whose untransmitted data size is smallest first. In addition, the sensor always transmits data at the time it receives more than the optimal energy $E^{*}$.

**Algorithm 1:** Minimize the total completion time.    **Input:** One enough long wireless energy receiving period $E_{1}=\left[0,+\infty \right]$, and *n* data blocks $\left\{D_{1},D_{2},\ldots ,D_{n}\right\}$, each $D_{i}=\langle a_{i},b_{i}\rangle$ denotes an online block of data with size $b_{i}$ arrives at time $a_{i}$.    **Output:** Find a solution to minimize the completion time of the data transmission. 1:Maintain a data list *L* that consists of the data that has not been completely transmitted.2:Whenever a block of data with size $b_{i}$ arrives, insert the data into the data list *L*. The data is always inserted into the position before the data block whose untransmitted data size is larger than $b_{i}$.3:Let the energy received at the sensor be *E*;4:Let the optimal energy point be $E^{*}$;5:**if**$E\geq E^{*}$then6:   Get a segment of data with size E−h from *L*, put it into a packet and transmit this packet; if the data size in *L* is less than E−h, then put all the data from *L* into the packet so as to use as more energy as possible.7:**else**8:   Continue to receive wireless energy until the received energy at the sensor gets to $E^{*}$;9:   Get a segment of data with size $E^{*}-h$ from *L*, put it into a packet and transmit this packet; if the data size in *L* is less than $E^{*}-h$, then put all the data from *L* into the packet so as to use as more energy as possible.10:**end if**

**Figure 5:**
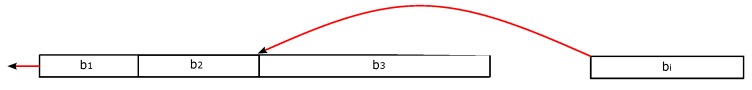
Illustration of Algorithm 1.

Figure 5 illustrates an example of Algorithm 1 where when a new block of data $b_{i}$ arrives, it will insert the new arriving data block into the data list *L* consisting of the data which has not been completely transmitted. The new arriving data block is always inserted before the data block which has larger untransmitted size. Let the non-completely transmitted data blocks in *L* have size $b_{1}$, $b_{2}$ and $b_{3}$. In addition, assume $b_{1}<b_{2}<b_{i}<b_{3}$, then the new arriving data block will be inserted into *L* before $b_{3}$ but after $b_{2}$. After inserting the new arriving data block, the sensor will get a segment of data from the list *L*, put it into a packet, and transmit this packet out. Note that the transmitted segment of data could consist of more than one block of data in *L*, and meanwhile the transmitted data could be part of a block of data in *L*.

**Lemma** **1.** *Transmitting the data block with smaller size first will not get longer total completion time.*

**Proof.**  Assume that there are only two blocks of data need to be transmitted. The data sizes are $b_{i}$ and $b_{j}$, respectively. Without loss of generality, let $b_{i}<b_{j}$. Let the transmission time of an optimal solution for data $b_{i}$ be $f(b_{i})$, and that for data $b_{j}$ be $f(b_{j})$. It is obvious that $f\left(b_{i}\right)<f\left(b_{j}\right)$. Let current time be *t*. Then, transmitting data $b_{i}$ first will get a completion time of $t+f\left(b_{i}\right)+t+f\left(b_{i}\right)+f\left(b_{j}\right)=2t+f\left(b_{i}\right)+f\left(b_{j}\right)+f\left(b_{i}\right)$, while transmit data $b_{j}$ first will get a completion of $2t+f\left(b_{i}\right)+f\left(b_{j}\right)+f\left(b_{j}\right)$. It is easy to find that transmitting data $b_{i}$ first has a smaller total completion time. □

**Figure 6:**
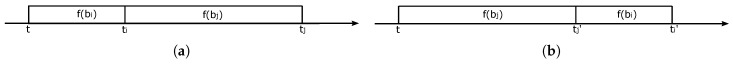
Different transmission order. (**a**) transmit $b_{i}$ first; (**b**) transmit $b_{j}$ first.

We use an example in Figure 6 to show the proof of Lemma 1. Assume that there are two data blocks with sizes of $b_{i}$ and $b_{j}$, respectively. If we transmit $b_{i}$ first, then the completion time for $b_{i}$ and $b_{j}$ are $t_{i}$ and $t_{j}$, respectively, as shown in Figure 6a, the total completion time is $t_{i}+t_{j}=t+f\left(b_{i}\right)+t+f\left(b_{i}\right)+f\left(b_{j}\right)$. However, if we transmit $b_{j}$ first, then the total completion time is $t_{j}^{\prime }+t_{i}^{\prime }=t+f\left(b_{j}\right)+t+f\left(b_{j}\right)+f\left(b_{i}\right)$ as shown in Figure 6b. Because $f\left(b_{i}\right)<f\left(b_{j}\right)$, it has $t_{i}+t_{j}\leq t_{j}^{\prime }+t_{i}^{\prime }$, which represents that transmitting $b_{i}$ first will not get a worse result.

Algorithm 1 is an online algorithm. It schedules the transmission without knowing the information of the future arriving data blocks. Online algorithms should be evaluated by competitive ratios that compare the result of the designed online algorithm with an optimal result. Competitive ratio is a metric to evaluate how a given online algorithm performs. People should design algorithms with as small a competitive ratio as possible.

**Theorem** **1.** *The competitive ratio of Algorithm 1 is $\frac{n+3}{n+1}$ where n is the number of arrived data blocks.*

**Proof.**  Define $E^{*}=h+b^{*}$ which is the optimal energy that the sensor should receive and then begin to transmit data. If the data size is less than $b^{*}$, then it will be transmitted by only one packet, thus the completion time will be increased by less than $\frac{E^{*}}{R}$ where *R* is the transmission rate. Algorithm 1 always transmits the data block whose untransmitted size is smaller first. This method is an optimal solution based on Lemma 1. If the size of a data block is less than $b^{*}$, then the algorithm will combine it with some of other data block to extend it to be $b^{*}$, and then transmit it. Otherwise, it indicates that there is no following data that can be used to combine into a packet. In this situation, transmitting the data block is optimal. Therefore, it can be easily found that the total completion time for *n* blocks of data is no less than $\frac{E^{*}}{R}\left(1+2+\ldots +n\right)=\frac{E^{*}}{R}\cdot \frac{(1+n)n}{2}$, where *n* is the number of data block whose size is larger than $b^{*}$. In addition, the online Algorithm 1 will increase the completion time for each block of data by at most $\frac{E^{*}}{R}$, thus the competitive ratio is represented as $\frac{\frac{E^{*}}{R}\cdot \frac{(1+n)n}{2}+\frac{E^{*}}{R}n}{\frac{E^{*}}{R}\cdot \frac{(1+n)n}{2}}=\frac{n+3}{n+2}$. It can be found that this competitive ratio is at most $\frac{3}{2}$ when *n* is close to 0. In addition, it trends to be 1 when *n* is large enough. □

## 4. Not Enough Wireless Energy Receiving Time

In some of the situations, the sensor cannot always receive enough wireless energy to transmit the arriving data. Therefore, we should consider the problem of how to determine the energy receiving and data transmission process so as to fully utilize the received wireless energy and transmit as much data as possible. In this paper, we do not consider how many data blocks are transmitted, but only consider how much data is transmitted. That is, data in different blocks have the same significance. This is important in applications that need to collect as much data as possible—for example, if a sensor needs to transmit a data block with a sequence of temperature values or images, and each temperature value or image is valuable. Thus, transmitting more data successfully is reasonable. We first consider a situation where there is only on period of time that the sensor can receive wireless energy, and then the scenario where there are multiple energy receiving periods is considered next.

### 4.1. Receiving Only One Period of Wireless Energy

Because the sensor can only receive one period (let it be [0,T]) of wireless energy, it should decided how to take full advantage of this period of time and transmit as much data as possible. If the sensor only uses this period of time to receive energy, then it may result in the received energy being much less than the sensor can receive in many shorter periods of time due to the energy receiving rate becoming slower and slower for capacitors.

We will discuss this case in two subproblems. The first subproblem is that there is only one block of data, but the data amount is extremely large so that the wireless energy the sensor can receive at most is not enough to transmit this block of data. The second subproblem is that there are many blocks of data arriving at different times, and the energy that can be received at most is not enough to transmit all the blocks of data as well.

#### 4.1.1. Only One Block of Data with an Extremely Large Size

When the wireless energy receiving time is limited, without loss of generality, let the energy receiving period be *T*. In such situations, the sensor should receive as much energy as possible so as to transmit more data. However, because the capacitor will receive energy slower and slower, it does not mean that receiving energy in the entire energy receiving period will get the energy to transmit more data. On the contrary, if the sensor receives a shorter period of time and then it begins to transmit data, it may receive more energy and transmit more data. However, transmitting in such way may also result in the transmitting time being wasted because, during the transmission time, the sensor cannot receive energy. In addition, transmitting in such way can also result in more transmission cost of packet headers. Therefore, we should determine when to receive wireless energy and when to transmit data so as to transmit a maximum amount of data.

**Figure 7:**
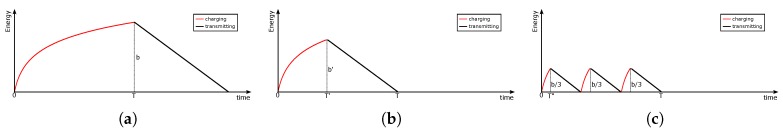
Find the maximum transmitted amount of data. (**a**) receive energy in the entire period of time *T*; (**b**) receive energy for a shorter period of time $T^{\prime }$ to transmit data; (**c**) receive energy for a more shorter period of time $T^{\prime \prime }$ to transmit data.

An example is shown in Figure 7, where the wireless energy receiving period is [0,T]. If the sensor receives energy in the entire period of time [0,T] as shown in Figure 7a, the sensor will receive energy that can transmit data with size *b*. If it receives energy during the time $\left[0,T^{\prime }\right]$ as shown in Figure 7b, and the time in $\left[T^{\prime },T\right]$ is used to transmit data, then it will receive energy to transmit data with size $b^{\prime }$ which is much less than *b*, and the time within $\left[T^{\prime },T\right]$ is wasted and it cannot receive energy. However, the sensor can also receive energy to transmit data with *b* when the energy receiving time is much shorter as shown in Figure 7c (In this example, we do not consider the transmission cost of packet header).

When there is a huge block of data whose size is sufficient large, then the entire wireless energy receiving period can be divided into multiple shorter energy receiving periods. Then, sensors can receive energy in each shorter energy receiving period and then begin to transmit a segment of data each time. Let the number of shorter energy receiving period be *m*, and the duration of each shorter receiving period be *t*, then the problem can be formulated as follows. The object of the problem is

(5) max{m·b},

subjects to:

(6) H(t)=h+b,

(7) $$T=m\cdot t+\left(m-1\right)\frac{h+b}{R}.$$

In this problem, *T* is the duration length of the wireless energy receiving period where the sensor can receive energy, *m* is the number of smaller packets that the sensor can transmit. *b* is the data size that should really be transmitted in each packet, and *h* is the size of the packet header. Let *R* be the transmission speed, if it can harvest enough energy for transmitting *m* smaller packets; then, during the entire energy receiving period [0,T], the sensor will receive wireless energy for *m* shorter periods of time *t*, and transmit m−1 packets, thus $T=m\cdot t+\left(m-1\right)\frac{h+b}{R}$, and the last packet transmission can be performed out of the energy receiving period [0,T]. Therefore, the total data amount that the sensor can transmit is m·b. The problem is to determine the period of time *t* that the sensor should take to receive energy and then begin to transmit data. After *m* shorter periods, the sensor will transmit data with total size m·b.

**Figure 8:**
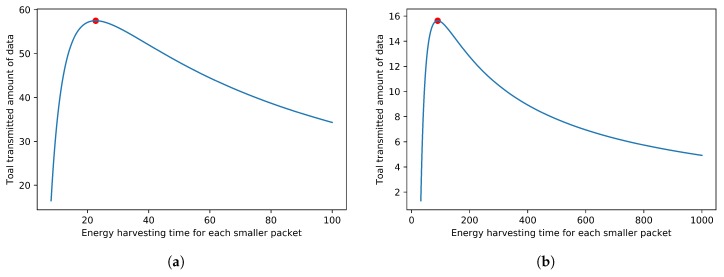
Find the maximum amount of transmitted data. (**a**) header = 3; (**b**) header = 5.

Because the wireless energy receiving time is limited, the problem is to maximize the total amount of data that could be transmitted. Again, we use an example in Figure 8 to show that there exists an optimal solution for this problem and the solution can be found through a binary search or Newton’s method. Figure 8 illustrates an example where the energy harvesting function is assumed to be $H\left(t\right)=\log_{2}\left(t+1\right)$, the duration length of the energy receiving period that the sensor can receive energy is T=1000, and the transmission rate is set to R=1. The energy harvesting function is the energy the capacitor will receive from the time the capacitor is initially empty. The data size is represented as the units of energy for simplicity. In addition, the transmission rate *R* denotes that it will take $\frac{h+b}{R}$ units of time to transmit a packet. In Figure 8, the *x*-axis is the time *t* that the sensor should take to receive wireless energy and then transmit part of the data immediately, and the *y*-axis is the total amount of data that the sensor would transmit. From Figure 8a,b, it can be easily found that the maximum total transmitted amount of data is at the position of the red points. The length of the packet header for Figure 8a is 3. The length of the packet header for Figure 8b is 5. For Figure 8a, the *x*-axis for the red point is about 22 and the *y*-axis is about 58, which means that, if the sensor always takes about 22 units of time to receive energy and then it begins to transmit data, the most amount of data that the sensor can transmit is about 58 when the entire wireless energy receiving period is [0,1000]. For Figure 8b, the packet header is 5, the *x*-axis for the red point is about 90, and the *y*-axis is about 16. Similarly, it denotes that, if the sensor always takes about 90 units of time to receive energy and then begins to transmit data, the data amount that the sensor can transmit at most is about 16 when the entire energy receiving period is [0,1000]. It can be found that there is a point that can get the optimal result as shown in the example. Again, the problem can be solved by Newton’s method, similar to what is stated in Section 3.1.

#### 4.1.2. Multiple Data Blocks Arrivng at Different Times

In this situation, we consider the situation where the data arrives at different times. The *i*th block of arriving data is represented as $\langle a_{i},b_{i}\rangle$ where $a_{i}$ is the arriving time and $b_{i}$ is the data size. Because data arrives at different times, the future data arriving information is not known in advance, the energy receiving period is limited, and the energy receiving rate changes over the time; therefore, it is hard to determine when to receive energy and when to transmit data.

Because there is only one period of limited energy receiving time [0,T], but the data arrives over time, if the sensor decides to receive wireless energy for a longer period of time so as to transmit more data, it may affected by the characteristic that the energy receiving rate is getting slower and slower. However, if the sensor decides to receive wireless energy for a shorter period of time and then begin to transmit data, it may fall into a bad situation where the data may arrive a very long time later. Therefore, a smart algorithm should be carefully designed so as to maximize the total transmitted amount of data while taking the trade-off into account.

Based on the previous section, we can find that there exists an optimal time point where the sensor receives wireless energy for a period of time $t^{*}$ until the received energy reaches the optimal energy $E^{*}$, and then begins to transmit data. In such a way, the sensor will transmit the most amount of data. We take use of this optimal energy receiving time point and design an online algorithm which is described in Algorithm 2.

**Algorithm 2:** Transmission algorithm when data arrives over time.    **Input:** One wireless energy receiving period $E_{1}=\left[0,T\right]$, and *n* data blocks $\left\{D_{1},D_{2},\ldots ,D_{n}\right\}$, each $D_{i}=\langle a_{i},b_{i}\rangle$ denotes an online block of data with size $b_{i}$ arrives at time $a_{i}$, 1≤i≤n.    **Output:** Schedule the data transmission to maximize the amount of transmitted data. 1:Maintain a data list *L* which consists of the data which has not been transmitted, any arriving data is appended to *L*;2:Whenever a block data arrives, check if the sensor is transmitting data or not and do the following process;3:**if** The sensor is not transmitting data **then**4:    **if** The energy in sensor $E\geq E^{*}$ **then**5:        **if** The data amount in *L* is greater than E−h **then**6:            Get a segment of data with size b=E−h from *L*, put it into a packet and transmit this packet;7:        **else**8:            Put all the data from *L* into a packet and transmit this packet;9:        **end if**10:    **else**11:     Continue to receive energy until the sensor receives energy $E^{*}$ or until the energy receiving period stops, and then begin to transmit data;12:    **end if**13:**else**14:    Wait until the transmission is completed;15:**end if** ——————————————————————————– 1:Whenever a packet is completely transmitted, start to receive energy until it reaches $E^{*}$, do the following process;2:**if** The data amount in *L* is greater than $b^{*}=E^{*}-h$ **then**3:    Get a segment of data with size $b^{*}$ from the list *L*, put it into a packet and transmit this packet;4:**else**5:    Receive energy until the data in *L* reaches to $b^{*}$ or until the energy receiving period stops, and then begin to transmit data;6:**end if**

In this algorithm, it maintains a data list *L* that consists of the data that arrived but has not been completely transmitted. When a block of data arrives, it is appended to *L* (It does not need to insert the arriving data block somewhere; this is because we just want to transmit as much data as possible, and it does not matter which block of data is transmitted). Then, the sensor checks if it is transmitting data at that time. If the sensor is transmitting data at that time, then the sensor will continue the transmission process. However, if the sensor is not transmitting data, then it should begin to transmit data only at the time when the energy received in the sensor is no less than $E^{*}$. When a packet is completely transmitted, then it should start to receive energy until the energy reaches $E^{*}$ and then begin to transmit data based on whether the amount of data in *L* is greater than $b^{*}$ or not. If the energy stored in the sensor is more than $E^{*}$ but the amount of data in *L* is less than $b^{*}$, then the sensor will continue to receive energy until some later blocks of data arrive and the amount of data in *L* becomes no less than $b^{*}$; otherwise, the energy receiving time stops and the sensor will start the transmission process.

**Theorem** **2.** *Algorithm 2 is a 2-competitive algorithm.*

**Proof.**  Let Alg be the solution of Algorithm 2 and Opt be an optimal solution. In our system, it assumes that the sensor cannot receive energy when it is transmitting data. There are only two cases that should be considered for this algorithm.

Case 1: the sensor has some energy, but it determines to receive energy. In this case, the amount of data in *L* is *b* and $b<b^{*}$; in other words, let the energy in the sensor be H(t); then, $H\left(t\right)<H\left(t^{*}\right)$. Howver, in an optimal solution Opt, the sensor would send the data in *L* immediately. The amount of data transmitted by Alg is $H(t^{*})$, and the amount of data transmitted by Opt is $H\left(t\right)+H\left(x^{\prime }\right)$ because some time is used to transmit data; therefore, $x^{\prime }<t^{*}-t$, and the competitive ratio is $\frac{H\left(t\right)+H\left(t^{*}-t\right)}{H(t^{*})}\leq 2$.

Case 2: the sensor has some energy but it determines to transmit data. In this case, the amount of data in *L* is greater than $b^{*}$ or the energy receiving period ends. Because there is only one energy receiving period, therefore, except that it is at the end of the energy receiving period, this transmission determination is optimal. This is because the energy receiving rate is getting slower and slower, thus the sensor should use the energy as quickly as possible. At the end of the energy receiving period, the data transmission determination may result in that the sensor will not receiving energy, but the unreceived energy is less than that the sensor received last time. Therefore, assume that the sensor can transmit *m* packets by Alg, then to avoid this situation, an optimal solution Opt can transmit as most m+1 packets. With loss of generality, for Alg, let the total size of first m−1 packets be *A* and the size of last packet be *B*, then the total amount of data that Alg can transmit is A+B. But Opt can transmit one packet more, then the total data size the Opt algorithm can transmit is at most A+B+B, therefore, it has $\frac{A+B+B}{A+B}$, when the data size A+B that Alg transmit is large enough, this ratio is close to 1, that is, the performance of Alg is close to an optimal solution Opt.

It can be found that the competitive ratio is the larger one of these two cases, thus, the competitive ratio of Algorithm 2 is 2. This completes the proof. □

### 4.2. Receive Multiple Periods of Wireless Energy

In some scenarios, there are many wireless energy receiving periods. This is reasonable because the wireless energy receiving time and the duration of the energy receiving period is unknown in many situations. Therefore, we should design an efficient algorithm to address this problem. This problem is more difficult because the energy receiving time and data arriving time is unpredictable in advance. Assume that the arriving data is much larger than the sensor can transmit; then, the problem is to transmit as much data as possible as well.

An online algorithm for this problem is provided in Algorithm 3. It is similar to Algorithm 2. The difference is that there are multiple wireless energy receiving periods, therefore, when the sensor successfully transmit a packet, it may not be within a energy receiving periods, then the sensor has to wait unit the next energy receiving period comes.

**Theorem** **3.** *The competitive ratio of Algorithm 3 is 2.*

**Proof.**  The proof is similar to the proof of Theorem 2. Let Alg be the solution of Algorithm 3 and Opt be an optimal solution. There are only two cases should be considered.

Case 1: It is the same as the proof of Theorem 2. The sensor has some energy but it determines to receive energy. In this case, the amount of data transmitted by Alg is $H(t^{*})$, and the amount of data transmitted by Opt is $H\left(t\right)+H\left(x^{\prime }\right)$ because some time is used to transmit data; therefore, $x^{\prime }<t^{*}-t$, the competitive ratio between Opt and Alg is $\frac{H\left(t\right)+H\left(t^{*}-t\right)}{H(t^{*})}\leq 2$.

Case 2: It is different from the proof of Theorem 2 that there are many energy receiving periods in this problem. In this case, the sensor has some energy, but it determines to transmit data. The amount of data in *L* is greater than $b^{*}$ or the energy receiving period ends. Let the energy in the sensor be H(t). Then, the amount of data transmitted by Alg is H(t) as well, and it takes $\frac{H(t)}{R}$ units of time to transmit data. However, in an optimal solution Opt, it may continue to receive energy and then begin to transmit data. The amount of data transmitted by this Opt is $H(t+\frac{H(t)}{R})$. Because of the characteristics that the energy receiving rate is getting slower and slower, the competitive ratio between Opt and Alg is $\frac{H(t+\frac{H(t)}{R})}{H(t)}<\frac{H\left(t\right)+H\left(\frac{H(t)}{R}\right)}{H(t)}\leq 2$. Otherwise, there exists a better $t^{{*}^{\prime }}$—this is a contract with the fact that $t^{*}$ is an optimal time point as shown in Figure 8.

Similarly, it can be found that the competitive ratio is the larger one of these two cases; thus, the competitive ratio of Algorithm 3 is 2. This completes the proof. □

**Algorithm 3:** Transmission algorithm when data and energy receiving time arrive over time.    **Input:**
*m* wireless energy receiving periods $\left\{E_{1},E_{2},\ldots ,E_{m}\right\}$, and *n* blocks of data arriving over time, where $E_{j}=\left[s_{j},e_{j}\right]$ represents the *j*th period of energy receiving period, and $D_{i}=\langle a_{i},b_{i}\rangle$ indicates an online block of data with size $b_{i}$ arrives at time $a_{i}$.    **Output:** Find a solution to maximize the amount of successfully transmitted data 1:Maintain a data list *L* which consists of the data which has not been transmitted, any arriving data is appended to *L*;2:Whenever a block data arrives, check if the sensor is transmitting data or not, and do the following process;3:**if** The sensor is not transmitting data **then**4:    **if** The energy in sensor $E\geq E^{*}$ **then**5:        **if** The data amount in *L* is greater than E−h **then**6:            Get a segment of data with size b=E−h from *L*, put it into a packet and transmit this packet;7:        **else**8:            Put all the data from *L* into a packet and transmit this packet;9:        **end if**10:    **else**11:            **while** the energy in the sensor is less than $E^{*}$ and the last energy receiving period does not stopped **do**12:                **if** The sensor is within an energy receiving period **then**13:                    Start to receive wireless energy until it reaches to $E^{*}$;14:                **else**15:                    Wait the next energy receiving period and the begin to receive wireless energy until it reaches to $E^{*}$, then it begins to transmit data;16:                **end if**17:            **end while**18:    **end if**19:**else**20:    Wait until the transmission is completed;21:**end if** ——————————————————————————– 1:Whenever a packet is completely transmitted, do the following process;2:**while** the energy in the sensor is less than $E^{*}$ and the last energy receiving period does not stopped **do**3:    **if** The sensor is within an energy receiving period **then**4:        Start to receive energy until it reaches to $E^{*}$;5:    **else**6:        Wait the next energy receiving period and the begin to receive energy until it reaches to $E^{*}$;7:    **end if**8:**end while**9:**if** The data amount in *L* is no less than $b^{*}=E^{*}-h$ **then**10:    Get a segment of data with size $b^{*}$ from the list *L*, put it into a packet and transmit this packet;11:**else**12:    Continue to receive energy until the data in *L* reaches to $b^{*}$ or until the last energy receiving period stops, and then it begins to transmit data;13:**end if**

## 5. Evaluations

In this section, we use simulation results to confirm that our algorithms have near optimal results. We have proved that our algorithm can have good results theoretically earlier in this paper. All the results of our online algorithms have bounded performance that is not too much worse than any optimal solutions. The simulations are implemented by C/C++ and Python. We randomly generate the data blocks and the energy receiving periods, and simulate the data transmission and energy receiving process to implement the algorithms. The competitive ratios that are used to evaluate the provided online algorithms are given as stated above. We compare our algorithm with the offline optimal algorithm and a simple method that always transmits the data block as entirely as possible. The optimal result is derived by assuming that all the information is known initially. The simple method is described as follows: if the sensor can receive enough energy in its capacitor to transmit a data block, then transmit this data block as a whole. Otherwise, receive energy until the capacitor is full, and completely use the received energy to transmit data. After some time, a big data block could be transmitted. We conduct two sets of simulations. In the first set of simulations, the energy receiving process is also generated. However, in the second set of simulations, the energy receiving process is from a real experiment.

**Figure 9:**
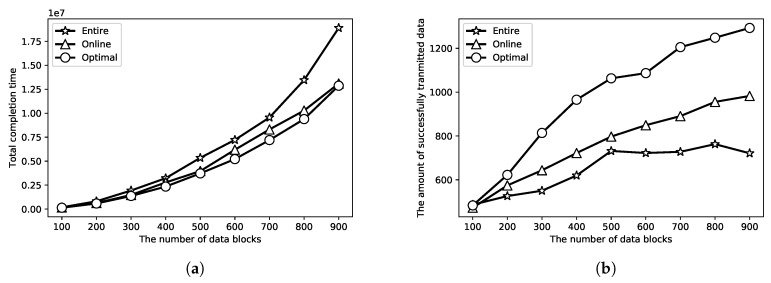
Result with simulated energy receiving process. (**a**) simulation for total completion time; (**b**) simulation for maximum successfully transmitted data amount.

In the first set of simulations, we compare the proposed online algorithms through randomly generated data. Each block of data arrives at a random time in [0,2000], and the data size is a random value in [1,20]. Each energy receiving period arrives at a random time in [0,2000], and two periods do not intersect with each other. We let the energy harvesting process be $H\left(t\right)=\omega \log_{x}\left(t+1\right)$, which is similar to the real energy receiving process. In addition, *x* is set as 2, and ω is set as 10. The maximum energy the capacitor can receive is set as 30. The speed of the data transmission is set as $\frac{1}{2}$. The performance of the proposed online algorithms is illustrated in Figure 9a,b which compare the online algorithm with the optimal algorithm and the simple algorithm. In Figure 9a,b, ‘Optimal’, ‘Online’, ‘Entire’ represents the optimal results, online results and the result of the simple algorithm that always transmits data block as entirely as possible, respectively. The performance of the total completion time is shown in Figure 9a where we simulate the performance from 100 to 900 blocks of data. It can be found in Figure 9a that the performance of the provided online algorithm is almost the same as the optimal solution, which confirms Theorem 1. In this result, the sensor can receive enough wireless energy to transmit all the randomly generated data blocks. The result of the total amount of successfully transmitted data is presented in Figure 9b where we simulate the result from 100 to 900 blocks of data again. However, the sensor cannot receive enough wireless energy to transmit all the data blocks. It can also be found in Figure 9b that the result of the presented online algorithm is not far away from the optimal result, and it is better than the simple algorithm. Thus, the provided algorithms in this paper have good performance.

**Figure 10:**
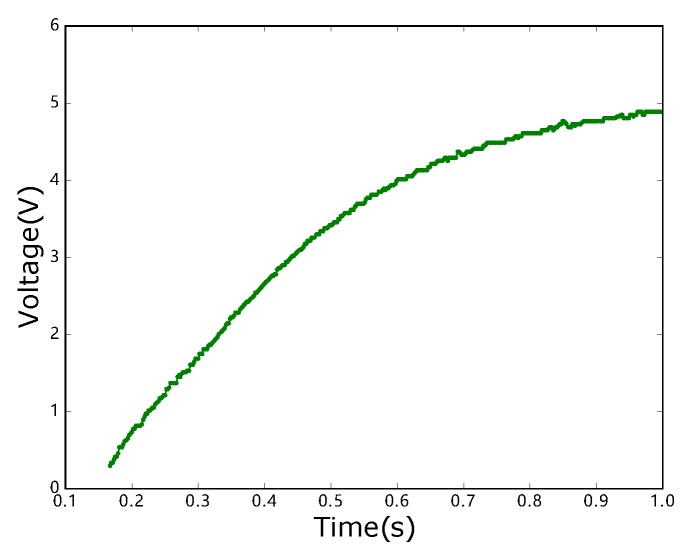
Wireless energy receiving process in a real device.

**Figure 11:**
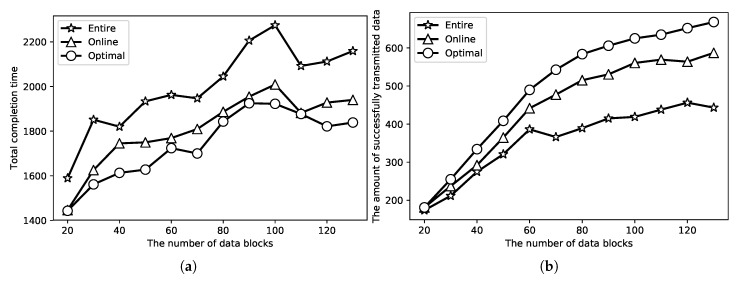
Result with real energy receiving process. (**a**) simulation for total completion time; (**b**) simulation for maximum successfully transmitted data amount.

In the next set of simulations, we compare the proposed online algorithm with the optimal solution and the simple algorithm through a real energy harvesting process which is from article [36] as shown in Figure 10. It can be found in Figure 10 that the energy receiving rate is becoming slower and slower. In this set of simulations, $E_{max}$ is the maximum value in Figure 10. Each data block arrives at random time in [0,100], and the data block size is random value in [0.2,1.5]. The speed of the data transmission is set as $\frac{1}{5}$. We just use the curve in this figure to simulate the energy receiving process. In addition, the number of data blocks is between 20 to 130 in both Figure 11a,b. Again, in Figure 11a,b, ‘Optimal’, ‘Online’, ‘Entire’ represents the optimal result, online result and the result of the simple algorithm, respectively. When the sensor can receive enough energy to transmit all the data blocks, then the result of total completion time is presented in Figure 11a. It can be found that the proposed online algorithm is similar to the optimal result; they do not differ not too much, and it is better than the simple algorithm. When sensors cannot receive enough energy to transmit all the data blocks, then the result of the maximum amount of successfully transmitted data is shown in Figure 11b. The performances of the online algorithm and the optimal result obey Theorem 2. Therefore, the presented online algorithms in this paper have good performance.

## 6. Conclusions

Data transmission with extra energy consumption from packet headers are considered in this paper. We take into account the characteristic of non-increasing energy receiving rate in sensors with capacitors to receive wireless energy, and study how it affects the data transmission process. We formulate two classes of problems. The first is that if the sensors can receive enough wireless energy, then we give an online algorithm to solve the minimum total completion time problem, and the competitive ratio of the given online algorithm is $\frac{n+3}{n+1}$. The second is that if the sensors cannot receive enough wireless energy, then we give an online algorithm to address the problem of maximum amount of successfully transmitted data, and the competitive ratio is 2. We simulate the presented online algorithms both in the generated and real energy receiving process. It can be found that our online algorithms have good performance in both sets of simulations by comparing them with the offline optimal algorithm and a simple algorithm.
